# Comprehensive DNA Adduct Analysis Reveals Pulmonary Inflammatory Response Contributes to Genotoxic Action of Magnetite Nanoparticles

**DOI:** 10.3390/ijms16023474

**Published:** 2015-02-04

**Authors:** Kousuke Ishino, Tatsuya Kato, Mamoru Kato, Tatsuhiro Shibata, Masatoshi Watanabe, Keiji Wakabayashi, Hitoshi Nakagama, Yukari Totsuka

**Affiliations:** 1 Division of Carcinogenesis and Prevention, National Cancer Center Research Institute, 1-1 Tsukiji 5-chome, Chuo-ku, Tokyo 104-0045, Japan; E-Mails: kishino@ncc.go.jp (K.I.); takatou1311@gmail.com (T.K.); hnakagam@ncc.go.jp (H.N.); 2 Division of Cancer Genomics, National Cancer Center Research Institute, 1-1 Tsukiji 5-chome, Chuo-ku, Tokyo 104-0045, Japan; E-Mails: mamkato@ncc.go.jp (M.K.); tashibat@ncc.go.jp (T.S.); 3 Division of Materials Science and Engineering, Graduate School of Engineering, Yokohama National University, Hodogaya-ku, Yokohama 240-8501, Japan; E-Mail: mawata@ynu.ac.jp; 4 Graduate Division of Nutritional and Environmental Sciences, University of Shizuoka, 52-1, Yada, Shizuoka 422-8526, Japan; E-Mail: kwakabayashi@u-shizuoka-ken.ac.jp

**Keywords:** magnetite nanoparticle, pulmonary inflammation, intratracheal instillation, DNA adductome

## Abstract

Nanosized-magnetite (MGT) is widely utilized in medicinal and industrial fields; however, its toxicological properties are not well documented. In our previous report, MGT showed genotoxicity in both *in vitro* and *in vivo* assay systems, and it was suggested that inflammatory responses exist behind the genotoxicity. To further clarify mechanisms underlying the genotoxicity, a comprehensive DNA adduct (DNA adductome) analysis was conducted using DNA samples derived from the lungs of mice exposed to MGT. In total, 30 and 42 types of DNA adducts were detected in the vehicle control and MGT-treated groups, respectively. Principal component analysis (PCA) against a subset of DNA adducts was applied and several adducts, which are deduced to be formed by inflammation or oxidative stress, as the case of etheno-deoxycytidine (εdC), revealed higher contributions to MGT exposure. By quantitative-LC-MS/MS analysis, εdC levels were significantly higher in MGT-treated mice than those of the vehicle control. Taken together with our previous data, it is suggested that inflammatory responses might be involved in the genotoxicity induced by MGT in the lungs of mice.

## 1. Introduction

Magnetite nanoparticles (MGT) have been widely utilized in medicinal and industrial fields [1]. Moreover, in medical applications, MGTs are widely used for magnetic resonance imaging as a contrast agent based on their good bio-compatibility [2,3]. With increasing utilization of MGT, it has been a concern whether MGTs are safe for humans or not. Hitherto, several reports describing MGT toxicity have been published [4,5,6,7,8,9,10,11,12,13], however, there is still controversy over reports regarding toxicity. Most investigations are focused on studying effects of MGTs on *in vitro* cellular viability, morphology and metabolism, or *in vivo* general toxicity on various organs with various administration routes of MGT (intraperitoneal, intratracheal or intravenous injection). Recently, we have reported genotoxic effects of MGTs using *in vitro* and *in vivo* assay systems, and clearly demonstrated that MGTs induce genotoxicity in both cultured mammalian cells and mice lungs instilled intratracheally [14,15,16]. Based on mutation spectra, histopathological evaluation, and oxidative- and lipid peroxide-related DNA adduct formations, it is suggested that inflammatory responses might contribute to the genotoxicity induced by MGT treatment [16].

It is well known that DNA adducts are considered to be triggers for induction of gene mutations [17,18,19,20,21]. In our previous report, MGT predominantly induced a C to T transition in mouse lungs [16]. Levels of 8-oxo-7,8-dihydro-2'-deoxyguanosine (8-oxodG) and heptano-etheno (Hɛ)-adducts were also elevated in lungs of mice exposed to MGT [16]. Although Hɛ-dC induces C to T transitions *in vitro* [22,23], the mechanisms of genotoxicity induced by MGT are not fully explained yet. Global discovery of DNA adducts in target organs would be useful information for exploring the mechanisms of genotoxicity.

Recently, Kanary *et al*. [24,25], established a method consisting of liquid chromatography followed by double tandem mass spectrometry for comprehensive analysis of DNA adducts in human and animal tissues. The basic strategy is designed to detect the neutral loss of a 2'-deoxyribose moiety [M + H; −116] from positively ionized 2'-deoxynucleoside adducts in multiple reaction ion monitoring mode (MRM) transmitting the precursor ion [M + H] ≥ daughter ion [M + H; −116] [24]. Using this method, hundreds of DNA adducts can be detected at once. Based on this strategy, we recently established comprehensive analysis of DNA adducts by using a UPLC-QTOF mass spectrometer. In this method, MS^E^ analysis was used to detect the neutral loss of a 2'-deoxyribose moiety [M + H; −116.04736]. Using this method, accurate mass values of precursor ions can be obtained, and this is an advantage for identification of chemical structures of DNA adducts. To identify the chemical structures of DNA adducts screened by adductome analysis, we have already made a list of DNA adducts, including *m*/*z* [M + H] values of precursor and daughter ions corresponding to more than 250 literature-based DNA adducts (see additional file 3). Moreover, the data obtained from *in vitro* model reactions such as oxidative stress, inflammation and alkylation are also included in this list. To clarify the mechanisms involved in genotoxicity induced by MGT, here, we examined the comprehensive DNA adduct analysis (DNA adductome analysis) of mice lungs exposed to MGT.

## 2. Results and Discussion

### 2.1. Comprehensive Analysis of DNA Adducts Induced by MGT (Nanosized-Magnetite) Treatment

Recently, we have reported that MGT clearly demonstrated genotoxicity in the lungs of *gpt* delta transgenic mice after intratracheal instillation [16]. As a result of mutation spectra analysis, most mutations induced by MGT occurred at G:C base pairs, and the prominent mutation types were a G:C to A:T transition followed by a G:C to T:A transversion [16].

**Figure 1:**
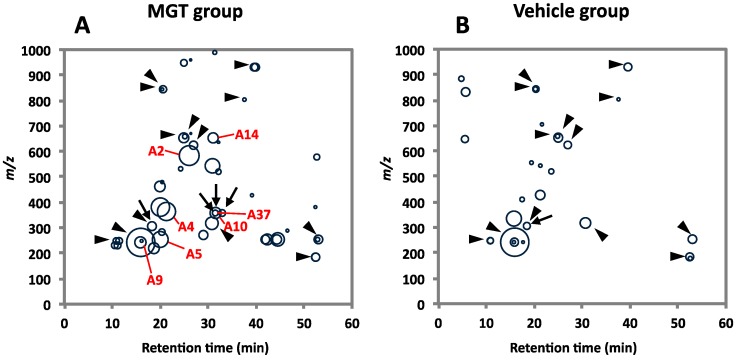
Comprehensive DNA adducts analysis. Map views of DNA adducts in lungs of mice with (**A**) or without MGT-exposure (**B**). Arrow heads indicate the DNA adducts observed in both MGT and vehicle groups, and arrows indicate the corresponding DNA adducts observed in *in vitro* model reactions, including oxidized arachidonic acid, oxidized linoleic acid or hydroxy radical with ctDNA (see additional file 1 and additional file 3). The 7 major contributors determined by PCA and RF analyses are indicated by A2, A4, A5, A9, A10, A14 and A37, respectively.

To investigate mechanisms of the induction of mutations in mouse lungs by MGT exposure, we performed comprehensive analysis of DNA adducts according to the methods described in “Experimental Section”. Totally, 30 and 42 types of DNA adducts were detected in the vehicle control and MGT-treated groups, respectively (Figure 1, and Additional File 1). Among them, 27 types of adducts were specific for the MGT-treated group, whereas 15 types of adducts overlapped between the MGT-treated and control groups.

**Figure 2:**
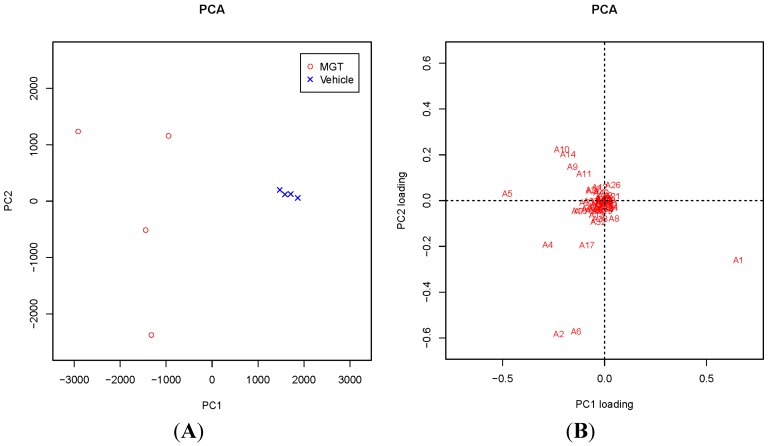
PCA scores and loading plots. (**A**) 2D PCA scores of DNA adducts obtained from adductome analysis. Principal components PC1 and PC2, which explains 74.25% of the total variance observed, discriminate the MGT-treated group from the vehicle control; (**B**) The PC1 and PC2 variable loading plots. Numbers A1–A53 represents DNA adducts observed in DNA adductome analysis.

**Figure 3:**
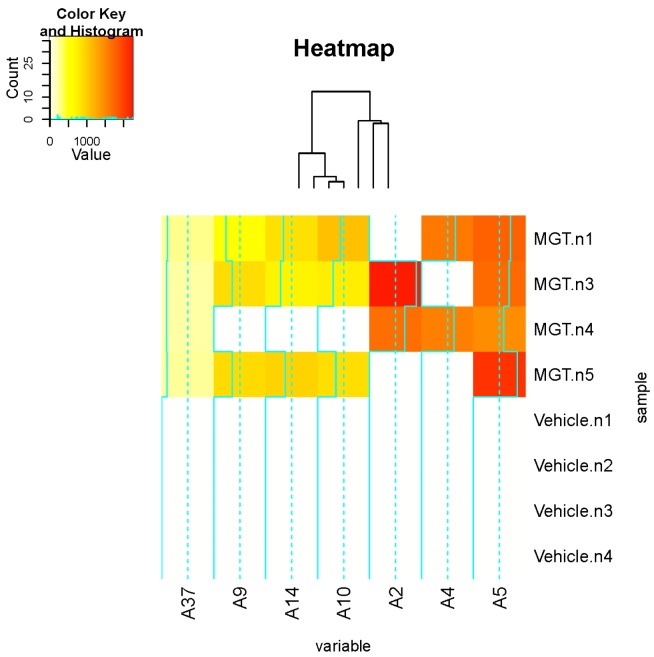
Heatmaps and clustering dendrogram. Hierarchical clustering was performed using 7 major contributors selected by PCA and RF analyses.

Principal component analysis (PCA) against a subset of DNA adducts observed in these data set was further applied and is shown in the 2D PCA scores plot (Figure 2A) and associated loadings plot (Figure 2B). A clear clustering of the data could be visualized according to vehicle control and MGT-treated mice (Figure 2A). The DNA adduct named A5 (*m*/*z* 252.11 [M + H] at *t*_R_ 20.1 min) had the highest contribution to MGT exposure based on its PCA significance. This was followed by DNA adducts named A4 (*m*/*z* 363.17 [M + H] at *t*_R_ 25.9 min), A10 (*m*/*z* 355.23 [M + H] at *t*_R_ 31.0 min), A14 (*m*/*z* 652.37 [M + H] at *t*_R_ 21.4 min) and A9 (*m*/*z* 243.12 [M + H] at *t*_R_ 31.0 min) revealed higher contribution to MGT exposure (Figure 2B). On the other hand, the DNA adduct named A1 demonstrated the highest contribution to the vehicle control. To confirm the results from PCA analysis, a random forest (RF) analysis of the DNA adductome profile data was also performed. The DNA adducts effectively separated the groups (vehicle *vs.* MGT) and are shown in the importance plot (Additional File 1). Several DNA adducts, including A5, A10 and A14, were the most important variables causing the clustering in both mean decrease in accuracy and mean decrease in Gini index (Additional File 1). A hierarchical clustering was analyzed on the dataset consisting of the DNA adducts diagnosed as highly contributing to MGT exposure, including A5 and A10, which were selected from both PCA and RF analyses. As shown in Figure 3, the heatmap for all contributors showed a clear separation of the MGT-treated group from the vehicle control. Among these, A5 was highly correlated with MGT treatment, whereas other contributors, such as A9, A14, A10, A2 and A4 seemed to not always correlate with MGT status. On the other hand, A37 also demonstrated a clear relation with MGT status, however the abundance was lower than that of A5.

### 2.2. Identification of DNA Adducts Correlated with MGT Treatment

To identify the chemical structure of DNA adducts detected as the “major contributors” to MGT status, we used the list of DNA adducts constructed by ourselves (Additional File 3). Firstly their values of *m*/*z* [M + H] were compared with known DNA adducts listed in Additional File 3. Among seven major contributors, A4, A5 and A9 indicated identical *m*/*z* values [M + H] for inflammation-related adducts, ammonium added butano-etheno-deoxyadenosine (BεdA-NH_3_, *m*/*z* 363.1816 [M + H]), etheno-deoxycytidine (εdC, *m*/*z* 252.0984 [M + H]) and 3-methyldeoxycytidine (3-medC, *m*/*z* 243.1213 [M + H]), respectively. In contrast to this, we could not find identical *m*/*z* values [M + H] for the remaining 4 contributors in the DNA adduct list. In order to clarify the formation mechanism of the remaining 4 contributors, A2, A10, A14 and A37, we prepared various *in vitro* model reactions, including oxidative stress and inflammation, and compared their *m*/*z* values [M + H] and *t*_R_ with each other. As a result, A10 (*m*/*z* 355.23 [M + H] at *t*_R_ 31.5 min) and A37 (*m*/*z* 356.24 [M + H] at *t*_R_ 31.4 min) were seen to correspond to one of the DNA adducts observed in the reaction mixture with oxidized-arachidonic acid (Additional File 1 and Additional File 2). No adducts having *m*/*z* 580.79 [M + H] at *t*_R_ 25.9 min (A2) and *m*/*z* 652.37 [M + H] at *t*_R_ 31.0 min (A14) could be seen in any of the *in vitro* model reactions. From these observations, it is suggested that inflammatory responses might exist in the mechanisms behind the increase in mutations by MGT treatment.

### 2.3. Confirmation of DNA Adducts Correlated with MGT Treatment

**Figure 4:**
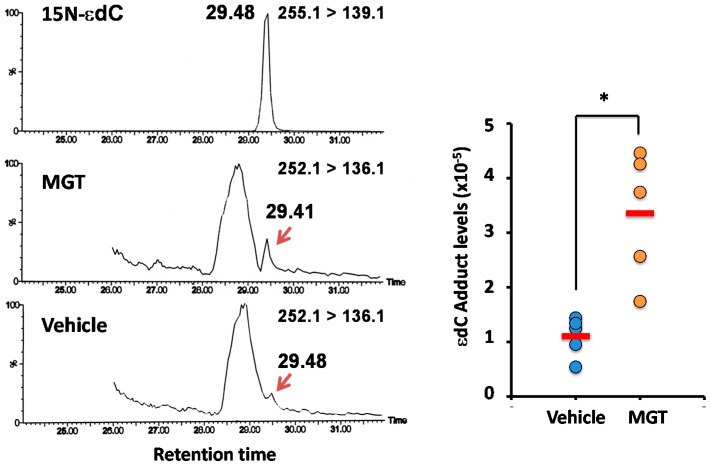
Quantitative Analysis of εdC by LC-MS/MS. εdC formation was induced by MGT exposure in the lungs of ICR mice. DNA was extracted from the lungs 24 h after intratracheal instillation of 0.2 mg per animal of MGT, and was digested enzymatically. Control samples were obtained from the lungs of mice given the vehicle for the same durations of MGT exposure. εdC were quantified by stable isotope dilution liquid chromatography-mass spectrometry (LC-MS/MS). Asterisk (*) indicates a significant difference (*p* < 0.05) from vehicle control (treatment with 0.05% (*v*/*v*) Tween-80) in the Student’s *t*-test.

In order to confirm the chemical structure of DNA adducts diagnosed as highly contributing to MGT status, we synthesized authentic 15N-εdC and analyzed it by quantitative LC-MS/MS apparatus (Waters 2795 LC system interfaced with a Quattro Ultima triple stage quadrupole MS, (Waters, Manchester, UK)). A peak with a 252.1 ≥ 136.1 transition corresponding to εdC, eluted at the same position as authentic 15N-εdC (255.1 ≥ 139.1), was observed in the lungs of both vehicle and MGT-treated mice (Figure 4). Levels of εdC were significantly higher in the MGT-treated group than those of the vehicle control (Figure 4).

εdC is produced from 4-hydroperoxy-2-nonenal via lipid peroxidation, and is known to be an inflammation-related adduct [22]. Since it has been reported that εdC is involved in C to T transitions using *in vitro* assay [23], it is likely that inflammatory responses might exist in the mechanisms behind the increase in mutations by MGT treatment. Although no data are available regarding the pulmonary inflammation generated by MGT single dose exposure in the present study, we have previously demonstrated that increasing oxidative stress and inflammation-related DNA adducts, including 8-oxodG and HεdC in the lungs of MGT-treated mice [16]. In addition, ROS production and overexpression of heme oxygenase-1, which mediates an anti-inflammatory effect, were clearly observed in MGT-exposed human lung epithelial cells, A549 [14]. Supporting our hypothesis, Park *et al.* [26], have been reported that single intratracheal instillation of magnetite increased the concentration of pro-inflammatory cytokines, such as TNF-α and IL-6, in the cells of bronchoalveolar lavage (BAL) fluid after 24 h exposure. Therefore, in the present study, it is reasonable to consider that inflammatory response evoked from the host reaction against foreign bodies, MGT, induce formation of inflammation-related DNA adducts, such as εdC and HεdC, which, being involved in C to T transitions, are more likely to contribute to genotoxicity observed in the lungs of MGT-exposed mice. Recently, several reports show that the mechanisms of (geno)toxicity induced by nanoparticles are suggested to be involved in macrophage stimulation [26,27,28,29,30,31,32,33]. Innate immune activation through Nalp3 inflammasomes has been suggested to play an important role in the pulmonary inflammation and fibrotic disorders of silicosis and asbestosis [31,32]. He *et al.* [29], demonstrated that multi walled carbon nanotubes (MWCNTs) directly induce inflammatory cytokines and chemokines, including TNF-α, IL-1β, IL-6, and MCP1 in murine macrophage cell line RAW264.7. Therefore, it is suggested that MGT can activate alveolar macrophage in the same way, then damage adjacent alveolar epithelial cells via cytokine and chemokine activation. In contrast, it has not been ruled out that direct toxicity against alveolar cells might be partly involved in induction of *in vivo* genotoxicity. It has been reported that MWCNTs damaged mitochondria to increase ROS production and cause toxicity against lung alveolar epithelial cells, A549 [29]. Similarly, we also have recently reported that MGT actually manifests cytotoxicity and clastogenicity in cultured mammalian cells [14,15]. Taken together, MGT elicits multiple events such as oxidative stress and inflammatory cytokine production, then leads to genotoxicity in mice lungs.

## 3. Experimental Section

### 3.1. Materials

MGT was purchased from Toda Industrial Co., Ltd. (Hiroshima, Japan), and this material was identical to those used in the *gpt* delta mouse study of Totsuka *et al.*, 2014 [16]. The declared primary particle size of MGT was 10.0 nm diameter around. The surface area was 125 m^2^/g (disclosed by Toda Industrial Co., Ltd.). Detailed information, such as particle appearance, dispersed diameter and zeta potential of MGT can be found in the previous report [16].

### 3.2. Chemicals

NucleaseP1 and HPLC grade methanol were purchased from Wako (Tokyo, Japan). Phosphodiesterase I was purchased from Worthington. Bovine spleen phosphodiesterase II, DNase I, Type I agarose, low melting point agarose, and Triton X-100 and bacterial alkaline phosphatase Type III (*E. coli*) were purchased from Sigma Co. (St. Louis, MO, USA). All other chemicals used were of analytical grade and purchased from Wako.

### 3.3. Animals

Male ICR mice (6 weeks old) were obtained from Japan SLC (Shizuoka, Japan). Animals were provided with food (CE-2 pellet diet, CLEA Japan, Inc., Tokyo, Japan) and tap water *ad libitum* and quarantined for one week. Mice were maintained under controlled conditions: 12 h light/dark cycle, 22 ± 2 °C room temperature, and 55% ± 10% relative humidity. The experiments were conducted according to the “Guidelines for Animal Experiments in the National Cancer Center” of the Committee for Ethics of Animal Experimentation of the National Cancer Center.

### 3.4. Analysis of DNA Adducts

For DNA adduct analyses, each group of 4 to 5 male ICR mice was intratracheally instilled with MGT at a single dose of 0.2 mg per animal, and sacrificed 24 h after MGT administration. Our previous study [16] demonstrated that *gpt* mutation frequency was significantly increased in mice lungs treated with multiple doses of 0.2 mg, but not in the 0.05 mg treatment group. In the DNA adduct formation analysis, even though singly treated with 0.2 mg of MGT, the levels of oxidative stress related DNA adducts were significantly increased. Therefore, we thought that a single dose of 0.2 mg MGT/animal was sufficient to analyze comprehensive DNA adduct analysis. Control samples were obtained from the lungs of mice given the vehicle. Mouse lung DNA was extracted and purified using a Gentra^®^ Puregene™ tissue kit (QIAGEN, Valencia, CA, USA). The protocol was performed according to the manufacturer’s instructions except that desferroxamine (final concentration: 0.1 mM) was added to all solutions to avoid the formation of oxidative adducts during the purification step. The extracted DNA was stored at −80 °C until analysis for DNA adductome analysis.

#### 3.4.1. Comprehensive Analysis of DNA Adducts (DNA Adductome Analysis)

Mouse lung DNA extracted from vehicle (*n* = 4) and MGT treated (*n* = 4) mice were enzymatically digested according to the method of Goodenough *et al*. [24], with some modifications. Briefly, internal standards (2',3'-dideoxyinosine and 2',3'-dideoxyguanosine) were added to the DNA solution prior to enzyme digestion, at 12.7 nM. The enzymatic digestion conditions are as follows; DNA (67 µg) in 5 mM Tris-HCl buffer (pH 7.4) employed DNase I (Type IV from bovine pancreas) for 3 h. Next, nuclease P1 (from *Penicillium citrinum*), 10 mM sodium acetate (pH 5.3, final 10 mM), and ZnCl_2_ (final 34 mM) were added, and incubated for a further 3 h at 37 °C. Alkaline phosphatase (from *E. coli*), phosphodiesterase I (20 U/mL in water) and Tris base (final 15.4 mM) were added last, for an additional 16–18 h at 37 °C. The sample was purified using Vivacon500^®^ (10 kDa molecular weight cut-off filters, Sartorius AG, Goettingen, Germany), then, the reaction mixture was centrifuged (4 °C, 10,000× *g*, 15 min) using Ultrafree^®^ (0.2 µm pore; Millipore Co., Billerica, MA, USA) and the filtrate was used for DNA adductome analysis.

LC-MS analyses were performed using a nanoACQUITY UPLC system (Waters, Milford, MA, USA) equipped with a Xevo QTOF mass spectrometer (Waters, Manchester, UK), instrumented with an electrospray ionization source (ESI) and controlled by MassLynx 4.1 software. Sample injection volumes of 4 μL each were separated on a ACQUITY UPLC BEH130 C18 column (1.7 µm, 1.0 mm i.d. × 150 mm) at a flow rate of 25 µL/min. The column temperature was set to 40 °C. Mobile phase A and B were water and methanol, respectively. Chromatographic separation was performed by a gradient elution as follows: 0–5 min, 1% B; 5–10 min, linear gradient to 10% B; 10–35 min, linear gradient to 80% B; 35–45 min, 80% B. MS parameters were set as follows: mass range scanned from 50 to 1000 with a scan duration of 0.5 s (1.0 s total duty cycle), capillary 3.7 kV, sampling cone 40 V, extraction cone 4 V, source temperature 125 °C, desolvation temperature 250 °C. Nitrogen gas was also used as the desolvation gas (flow 800 L/h) and cone gas (30 L/h). All data were collected in positive ion mode. MS^E^ analysis was performed on the mass spectrometer set at 3 V for low collision energy and ramp of 10–25 V for high collision energy during the acquisition cycle. A cone voltage of 20 V was used. LockMass parameters were set as following: capillary 3.0 kV, sample cone 40 V, collision energy 21 V.

Relative peak intensity of each potential DNA adduct was calculated as previously described [33]. The relative peak intensity was plotted as a bubble chart in which the horizontal axis was retention time and the vertical axis was *m*/*z*. DNA obtained from normal human fetal fibroblast cell line, TIG-3, with internal standard was used as a reference [33].

#### 3.4.2. Data Processing

The raw data files obtained from LC/MS runs were analyzed using MassLynx v4.1 and MarkerLynx 4.1 software (Waters). The application detects, integrates, and normalizes the intensities of the peaks to the sum of peaks within the sample. The resulting multivariate dataset consisting of the peak number (based on the retention time and *m*/*z*), sample name, and the normalized peak intensity was analyzed by S-plot analysis using SIMCA-P+ 11.5 (Umetrics AB, Umea, Sweden). The method parameters were as follows: Mass tolerance = 0.05 Da, Apex Track Parameters: Peak width at 5% height (seconds) = 15/Peak-to-peak baseline noise = 50, Apply smoothing = Yes, Collection Parameters: Intensity threshold (counts) = 100/Mass window = 0.05/Retention time window = 0.10, Noise elimination level = 6, Deisotope data = Yes.

#### 3.4.3. *In Vitro* Modification of DNA

The DNA modification derived from oxidation of unsaturated fatty acids was performed by incubating calf thymus DNA (ctDNA, 1 mg/mL, Sigma, Steinheim, Germany) with 20 mM unsaturated fatty acids including arachidonic acid (Sigma) and linoleic acid (Sigma) in the presence of 75 µM CuSO_4_ (Wako) and 1.5 mM ascorbic acid (Wako). The DNA modification related to oxidative stress was formed from ctDNA and 10 mM hydrogen peroxide (Wako) in the presence of 1 mM CuSO_4_ and 1 mM ascorbic acid in 1 mL of 500 mM sodium phosphate buffer, pH 7.4 for 24 h, in atmospheric oxygen at 37 °C. The reaction was terminated by the addition of 1 mM butylated hydroxytoluene (Wako) and 100 µM diethylenetriaminepentaacetic acid (Wako).

#### 3.4.4. Confirmation of εdC

Mouse lung DNA (40 μg) extracted from vehicle (*n* = 5) and MGT treated (*n* = 5) mice was enzymatically digested, and εdC was analyzed and quantified by the Waters 2795 LC system (Waters, Manchester, UK) interfaced with a Quattro Ultima triple stage quadrupole MS (Waters) using the same procedure previously described [34]. Authentic 15N-εdC was kindly provided by Dr. Yoshitaka Matsushima (Tokyo University of Agriculture) synthesized according to previously published methods [35]. The multiple reaction monitoring transitions were monitored; each cone voltage and collision energy used were εdC [252.1 ≥ 136.1, 35 V, 10 eV].

### 3.5. Statistical Analysis

PCA and RF analyses were used for modeling comprehensive analysis of DNA adducts (DNA adductome analysis). All the calculations were performed using statistical package R. In RF, we confirmed that the out-of-bag misclassification rate was saturated at 0% with 100,000 generated trees with two variables. The data were statistically compared with the corresponding solvent control using the Student’s *t* test for DNA adduct formation. The data were compared with the corresponding solvent control using the F test before application of the Student’s *t* test. If the F test evaluation showed an unequal variance, the *p* value was determined using the Welch’s *t* test.

## 4. Conclusions

We have demonstrated that MGTs induce inflammation-related DNA adduct formation in mouse lungs by using comprehensive analysis of DNA adducts, DNA adductome analysis. In PCA analysis, εdC was detected as the “major contributor” to MGT status. Due to the inducible base, the exchange pattern of εdC has been reported to be a C to T transition [], being the predominant mutational pattern detected in mouse lungs exposed to MGT [16]. Therefore, it is suggested that inflammatory responses lead to inflammation-related DNA adduct formations, such as εdC, and this might contribute to the genotoxicity in mouse lungs induced by MGT treatment.

## Figures and Tables

**Additional File 2:**
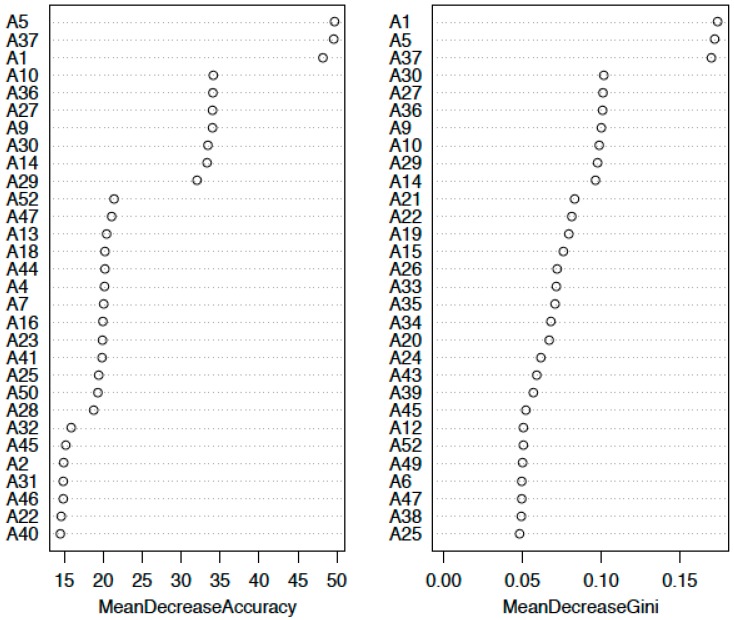
RF Dotchart of variable importance for vehicle control *vs.* MGT-treated group.

## Appendix

**Additional File 1:** List of the data set obtained from DNA adductome analysis.

Peak No.	RT (min)	*m*/*z*	Area Mean ± SD		*p*-Value (*t* Test)	Fold Change
			MGT	Vehicle		
1	16.0	242.11	4867 ± 722	4938 ± 2414	0.485	0.99
2	25.9	580.79	2389 ± 837	-	-	-
3	21.4	363.17	2016 ± 850	-	-	-
4	20.1	252.11	1543 ± 375	-	-	-
5	31.0	543.33	1273 ± 732	-	-	-
6	44.4	252.11	1099 ± 669	-	-	-
7	30.9	317.17	957 ± 125	676 ± 363	0.294	1.41
8	16.0	243.12	775 ± 112	-	-	-
9 ^a^	31.5	355.23	758 ± 221	-	-	-
10	18.8	219.11	703 ± 819	-	-	-
11	20.0	463.73	677 ± 504	-	-	-
12	31.0	652.37	632 ± 249	-	-	-
13	25.0	655.40	591 ± 188	493 ± 39	0.106	1.20
14	28.9	273.18	491 ± 205	-	-	-
15	41.8	252.11	471 ± 111	-	-	-
16 ^c^	18.4	308.13	468 ± 129	293 ± 27	0.010	1.59
17	53.0	252.11	458 ± 182	455 ± 162	0.447	1.03
18	52.5	181.98	415 ± 167	387 ± 156	0.425	1.07
19	26.9	622.79	411 ± 132	349 ± 110	0.287	1.18
20	39.7	928.61	379 ± 137	384 ± 83	0.490	0.99
21	20.2	841.27	317 ± 310	259 ± 179	0.452	1.23
22	11.0	230.11	295 ± 82	-	-	-
23 ^b^	32.9	360.21	269 ± 98	-	-	-
24	25.0	946.44	265 ± 154	-	-	-
25	20.2	284.13	252 ± 34	-	-	-
26	10.9	250.08	252 ± 70	264 ± 135	0.176	0.95
27	52.5	575.30	213 ± 49	-	-	-
28 ^a^	31.4	356.24	207 ± 50	-	-	-
29	32.0	517.69	174 ± 79	-	-	-
30	25.0	656.40	136 ± 33	115 ± 11	0.393	1.19
31	24.2	530.75	117 ± 38	-	-	-
32	52.8	253.11	108 ± 83	-	-	-
33	31.4	988.64	98 ± 23	-	-	-
34	37.5	800.44	70 ± 27	61 ± 13	0.451	1.14
35	39.1	429.25	58 ± 10	-	-	-
36	16.0	245.23	53 ± 27	-	-	-
37	52.4	382.20	50 ± 10	-	-	-
38	20.4	842.28	47 ± 41	123 ± 56	0.367	0.38
39	32.1	633.74	33 ± 25	-	-	-
40	32.1	634.10	33 ± 4	-	-	-
41	26.3	961.47	26 ± 14	-	-	-
42	26.4	667.80	19 ± 9	-	-	-

^a^ This peak overlapped with one of the adducts produced by reaction of oxidized arachidonic acid with ctDNA (data not shown); ^b^ This peak overlapped with one of the adducts produced by reaction of hydroxy radical with ctDNA (data not shown); ^c^ This peak overlapped with one of the adducts produced by reaction of oxidized linoleic acid with ctDNA (data not shown).

**Additional File 3:** Information of the authentic DNA adducts.

Adduct	Precurser (M + H)	Product (Deoxyribose Loss)	Ref.	Na 22.9898	K 39.0983	NH_3_ 18.0379
5-MedC	242.1140	126.0666	-	264.0960	280.2045	259.1441
dU	229.0824	113.0350	[36]	251.0644	267.1729	246.1125
dI	253.0936	137.0462	[36]	275.0756	291.1841	270.1237
dX	269.0886	153.0412	[36]	291.0706	307.1791	286.1187
dO	269.0886	153.0412	[36]	291.0706	307.1791	286.1187
8-Oxo-dG	284.0994	168.0520	[37]	306.0814	322.1899	301.1295
Sp	300.0944	184.0470	[38]	322.0764	338.1849	317.1245
Gh	274.1151	158.0677	[38]	296.0971	312.2056	291.1452
Iz	229.0937	113.0463	[38]	251.0757	267.1842	246.1238
Oz	247.1042	131.0568	[39]	269.0862	285.1947	264.1343
FapyG	286.1151	170.0677	[39]	308.0971	324.2056	303.1452
Oxa	249.0723	133.0249	[38]	271.0543	287.1628	266.1024
Cyclo-dG	266.0889	150.0415	[40]	288.0709	304.1794	283.1190
Cyanuric acid	246.0726	130.0252	[38]	268.0546	284.1631	263.1027
CAC	288.0944	172.0470	[41]	310.0764	326.1849	305.1245
HICA	277.0672	161.0198	[41]	299.0492	315.1577	294.0973
8-OH-dA	268.1046	152.0572	[39]	290.0866	306.1951	285.1347
2-OH-dA	268.1046	152.0572	[42]	290.0866	306.1951	285.1347
FapydA	270.1202	154.0728	[39]	292.1022	308.2107	287.1503
Cyclo-dA	250.0940	134.0466	[39]	272.0760	288.1845	267.1241
5-OHdC	244.0933	128.0459	[39]	266.0753	282.1838	261.1234
5-HmdU	259.0930	143.0456	[39]	281.0750	297.1835	276.1231
FodU	257.0773	141.0299	[39]	279.0593	295.1678	274.1074
Tg	277.1036	161.0562	[39]	299.0856	315.1941	294.1337
d(G[8–5]C)	555.1353	439.0879	[43]	515.1615	531.2700	510.2096
d(G[8–3]T)	508.1792	392.1318	[44]	530.1612	546.2697	525.2093
d(G[8–5m]T)	508.1792	392.1318	[45]	530.1612	546.2697	525.2093
εdA	276.1096	160.0622	[39]	298.0916	314.2001	293.1397
εdC	252.0984	136.0510	[39]	274.0804	290.1889	269.1285
ε5mdC	266.1100	150.0626	[46]	288.0920	304.2005	283.1401
εdG	292.1046	176.0572	[39]	314.0866	330.1951	309.1347
M1dG	304.1046	188.0572	[39]	326.0866	342.1951	321.1347
5,6-dihydro-M1dG	306.1202	190.0728	[47]	328.1022	344.2107	323.1503
PdG	308.1359	192.0885	[39]	330.1179	346.2264	325.1660
6-oxo-M1dG	320.0995	204.0521	[48]	342.0815	358.1900	337.1296
MDA-dA	306.1202	190.0728	[49]	328.1022	344.2107	323.1503
MDA-dC	282.1090	166.0616	[49]	304.0910	320.1995	299.1391
8-OH-PdG	324.1307	208.0833	[50]	346.1127	362.2212	341.1608
6-OH-PdG	324.1307	208.0833	[50]	346.1127	362.2212	341.1608
propano-dA	308.1359	192.0885	[51]	330.1179	346.2264	325.1660
propano-dC	286.1403	170.0929	[51] *	308.1223	324.2308	303.1704
propano-5MedC	300.1560	184.1086	[51] *	322.1380	338.2465	317.1861
FDP-dG	362.1465	246.0991	[52] *	384.1285	400.2370	379.1766
α-Me-γ-OH-PdG (R- or S-α-Me-γ-OH-CRA-dG)	338.1464	222.0990	[50]	360.1284	376.2369	355.1765
Croton-dA	322.1516	206.1042	[50] *	344.1336	360.2421	339.1817
Croton-dC	300.1560	184.1086	[50] *	322.1380	338.2465	317.1861
Croton-5MedC	314.1717	198.1243	[50] *	336.1537	352.2622	331.2018
ICL-RD	589.2483	473.2009	[53]	611.2303	627.3388	606.2784
ICL-R	587.2326	471.1852	[53]	609.2146	625.3231	604.2627
ICL-S	587.2326	471.1852	[53]	609.2146	625.3231	604.2627
Hexanoyl-dG	366.1777	250.1303	[54] *	388.1597	404.2682	383.2078
Hexenal-dG	366.1777	250.1303	[55]	388.1597	404.2682	383.2078
HNE-dG	424.2196	308.1722	[56]	446.2016	462.3101	441.2497
HNE-dA	408.2248	292.1774	[56] *	430.2068	446.3153	425.2549
HNE-dC	386.2292	270.1818	[56] *	408.2112	424.3197	403.2593
HNE-5MedC	400.2449	284.1975	[56] *	422.2269	438.3354	417.2750
HεdG	404.1933	288.1459	[57]	426.1753	442.2838	421.2234
HεdA	388.1984	272.1510	[57]	410.1804	426.2889	405.2285
HεdC	364.1872	248.1398	[57]	386.1692	402.2777	381.2173
HεMedC	378.2029	262.1555	[57] *	400.1849	416.2934	395.2330
BεdG	362.1464	246.0990	[58]	384.1284	400.2369	379.1765
BεdA	346.1515	230.1041	[58] *	368.1335	384.2420	363.1816
BεdC	322.1402	206.0928	[58]	344.1222	360.2307	339.1703
BεMedC	336.1559	220.1085	[58]	358.1379	374.2464	353.1860
CHPdG	460.2196	344.1722	[59] *	482.2016	498.3101	477.2497
CHPdA	444.2247	328.1773	[59] *	466.2067	482.3152	461.2548
CHPdC	420.2134	304.1660	[59] *	442.1954	458.3039	437.2435
CPPdG	404.1570	288.1096	[59] *	426.1390	442.2475	421.1871
CPPdA	388.1621	272.1147	[59] *	410.1441	426.2526	405.1922
CPPdC	364.1508	248.1034	[59] *	386.1328	402.2413	381.1809
CEPdG	390.1413	274.0939	[59] *	412.1233	428.2318	407.1714
CEPdA	374.1464	258.0990	[59] *	396.1284	412.2369	391.1765
CEPdC	350.1352	234.0878	[59] *	372.1172	388.2257	367.1653
*N*^6^-HmdA	282.1202	166.0728	[60]	304.1022	320.2107	299.1503
*N*^6^-MedA	266.1253	150.0779	[61]	288.1073	304.2158	283.1554
*N*^2^-Ethylidene-dG	294.1202	178.0728	[62]	316.1022	332.2107	311.1503
*N*^2^-ethyl-dG	296.1359	180.0885	[62]	318.1179	334.2264	313.1660
1-medA	268.1409	152.0935	[38]	290.1229	306.2314	285.1710
3-medC	243.1213	127.0739	[38]	265.1033	281.2118	260.1514
*N*^2^-CMdG	326.1100	210.0626	[63]	348.0920	364.2005	343.1401
Glyoxal-dA	310.1152	194.0678	[63]	332.0972	348.2057	327.1453
Glyoxal-dC	288.1196	172.0722	[63]	310.1016	326.2101	305.1497
Glyoxal-5MedC	302.1353	186.0879	[63] *	324.1173	340.2258	319.1654
*N*^2^-CEdG	340.1257	224.0783	[64]	362.1077	378.2162	357.1558
8-Cl-dG	302.0656	186.01824	[65]	324.0476	340.1561	319.0957
8-Cl-dA	286.0707	170.02334	[65]	308.0527	324.1612	303.1008
5-Cl-dC	262.0594	146.01204	[65]	284.0414	300.1499	279.0895
8-Br-dG	346.0151	229.96774	[66]	367.9971	384.1056	363.0452
8-Br-dA	330.0202	213.97284	[66] *	352.0022	368.1107	347.0503
5-Br-dC	306.0089	189.96154	[67]	327.9909	344.0994	323.039

Na: sodium added form; K: potassium added form; NH_3_: ammonium added form; *: Expected *m*/*z* calculated by Symyx Draw 4.0 software (Accelrys Inc., San Diego, CA, USA).

## References

[1] Cabreraa L., Gutierreza S., Menendezb N., Moralesc M.P., Herrasti P. (2008). Magnetite nanoparticles: Electrochemical synthesis and characterization. Electrochim. Acta.

[2] Jin R., Lin B., Li D., Ai H. (2014). Superparamagnetic iron oxide nanoparticles for MR imaging and therapy: Design considerations and clinical applications. Curr. Opin. Pharmacol..

[3] Felton C., Karmakar A., Gartia Y., Ramidi P., Biris A.S., Ghosh A. (2014). Magnetic nanoparticles as contrast agents in biomedical imaging: Recent advances in iron- and manganese-based magnetic nanoparticles. Drug Metab. Rev..

[4] Guichard Y., Schmit J., Darne C., Gaté L., Goutet M., Rousset D., Rastoix O., Wrobel R., Witschger O., Martin A. (2012). Cytotoxicity and genotoxicity of nanosized and microsized titanium dioxide and iron oxide particles in Syrian hamster embryo cells. Ann. Occup. Hyg..

[5] Aranda A., Sequedo L., Tolosa L., Quintas G., Burello E., Castell J.V., Gombau L. (2013). Dichloro-dihydro-fluorescein diacetate (DCFH-DA) assay. A quantitative method for oxidative stress assessment of nanoparticle-treated cells. Toxicol. Vitro.

[6] Singh N., Jenkins G.J., Asadi R., Doak S.H. (2010). Potential toxicity of superparamagnetic iron oxide nanoparticles (SPION). Nano Rev..

[7] Ramesh V., Ravichandran P., Copeland C.L., Gopikrishnan R., Biradar S., Goornavar V., Ramesh G.T., Hall J.C. (2012). Magnetite induces oxidative stress and apoptosis in lung epithelial cells. Mol. Cell Biochem..

[8] Könczöl M., Ebeling S., Goldenberg E., Treude F., Gminski R., Gieré R., Grobéty B., Rothen-Rutishauser B., Merfort I., Mersch-Sundermann V. (2011). Cytotoxicity and genotoxicity of size-fractionated iron oxide (magnetite) in A549 human lung epithelial cells: Role of ROS, JNK, and NF-κB. Chem. Res. Toxicol..

[9] Karlsson H.L., Gustafsson J., Cronholm P., Möller L. (2009). Size-dependent toxicity of metal oxide particles—A comparison between nano- and micrometer size. Toxicol. Lett..

[10] Ma P., Luo Q., Chen J., Gan Y., Du J., Ding S., Xi Z., Yang X. (2012). Intraperitoneal injection of magnetic Fe_3_O_4_-nanoparticle induces hepatic and renal tissue injury via oxidative stress in mice. Int. J. Nanomed..

[11] Weissleder R., Stark D.D., Engelstad B.L., Bacon B.R., Compton C.C., White D.L., Jacobs P., Lewis J. (1989). Superparamagnetic iron oxide: Pharmacokinetics and toxicity. Am. J. Roentgenol..

[12] Singh N., Jenkins G.J., Nelson B.C., Marquis B.J., Maffeis T.G., Brown A.P., Williams P.M., Wright C.J., Doak S.H. (2012). The role of iron redox state in the genotoxicity of ultrafine superparamagnetic iron oxide nanoparticles. Biomaterials.

[13] Szalay B., Tátrai E., Nyírő G., Vezér T., Dura G. (2012). Potential toxic effects of iron oxide nanoparticles in *in vivo* and *in vitro* experiments. J. Appl. Toxicol..

[14] Watanabe M., Yoneda M., Morohashi A., Okamoto D., Sato A., Kurioka D., Hirokawa H., Shiraishi T., Kawai K., Kasai K. (2013). Effects of Fe_3_O_4_-based magnetic nanoparticles on A549 cells. Int. J. Mol. Sci..

[15] Kawanishi M., Ogo S., Ikemoto M., Totsuka Y., Ishino K., Wakabayahsi K., Yagi T. (2013). Genotoxicity and reactive oxygen species production induced by magnetite nanoparticles in mammalian cells. J. Toxicol. Sci..

[16] Totsuka Y., Ishino K., Kato T., Goto S., Tada Y., Nakae D., Watanabe M., Wakabayashi K. (2014). Magnetite nanoparticles induce genotoxicity in the lungs of mice via inflammatory response. Nanomaterials.

[17] Kew M.C. (2013). Aflatoxins as a cause of hepatocellular carcinoma. J. Gastrointest. Liver Dis..

[18] Hollstein M., Moriya M., Grollman A.P., Olivier M. (2013). Analysis of TP53 mutation spectra reveals the fingerprint of the potent environmental carcinogen, aristolochic acid. Mutat. Res..

[19] Hecht S.S. (2012). Lung carcinogenesis by tobacco smoke. Int. J. Cancer.

[20] Khalili H., Zhang F.J., Harvey R.G., Dipple A. (2000). Mutagenicity of benzo[*a*]pyrene-deoxyadenosine adducts in a sequence context derived from the *p53* gene. Mutat. Res..

[21] Scholdberg T.A., Nechev L.V., Merritt W.K., Harris T.M., Harris C.M., Lloyd R.S., Stone M.P. (2005). Mispairing of a site specific major groove (2S,3S)-N^6^-(2,3,4-trihydroxybutyl)-2'-deoxyadenosyl DNA Adduct of butadiene diol epoxide with deoxyguanosine: Formation of a dA(anti)·dG(anti) pairing interaction. Chem. Res. Toxicol..

[22] Pollack M., Yang I.Y., Kim H.Y., Blair I.A., Moriya M. (2006). Translesion DNA Synthesis across the heptanone-etheno-2'-deoxycytidine adduct in cells. Chem. Res. Toxicol..

[23] Yang I.Y., Hashimoto K., de Wind N., Blair I.A., Moriya M. (2009). Two distinct translesion synthesis pathways across a lipid peroxidation-derived DNA adduct in mammalian cells. J. Biol. Chem..

[24] Kanaly R.A., Hanaoka T., Sugimura H., Toda H., Matsui S., Matsuda T. (2006). Development of the adductome approach to detect DNA damage in humans. Antioxid. Redox Signal..

[25] Matsuda T., Tao H., Goto M., Yamada H., Suzuki M., Wu Y., Xiao N., He Q., Guo W., Cai Z. (2013). Lipid peroxidation-induced DNA adducts in human gastric mucosa. Carcinogenesis.

[26] Park E.J., Kim H., Kim Y., Yi J., Choi K., Park K. (2010). Inflammatory responses may be induced by a single intratracheal instillation of iron nanoparticles in mice. Toxicology.

[27] Hsiao J.K., Weng T.I., Tai M.F., Chen Y.F., Wang Y.H., Yang C.Y., Wang J.L., Liu H.M. (2009). Cellular behavior change of macrophage after exposure to nanoparticles. J. Nanosci. Nanotechnol..

[28] Xia T., Kovochich M., Liong M., Zink J.I., Nel A.E. (2008). Cationic polystyrene nanosphere toxicity depends on cell-specific endocytic and mitochondrial injury pathways. ACS Nano.

[29] He X., Young S.H., Schwegler-Berry D., Chisholm W.P., Fernback J.E., Ma Q. (2011). Multiwalled carbon nanotubes induce a fibrogenic response by stimulating reactive oxygen species production, activating NF-κB signaling, and promoting fibroblast-to-myofibroblast transformation. Chem. Res. Toxicol..

[30] Kasper J.L., Hermanns M.I., Bantz C., Maskos M., Stauber R., Pohl C., Unger R.E., Kirkpatrick J.C. (2011). Inflammatory and cytotoxic responses of an alveolar-capillary coculture model to silica nanoparticles: Comparison with conventional monocultures. Part. Fibre Toxicol..

[31] Dostert C., Pétrilli V., van Bruggen R., Steele C., Mossman B.T., Tschopp J. (2008). Innate immune activation through Nalp3 inflammasome sensing of asbestos and silica. Science.

[32] Cassel S.L., Eisenbarth S.C., Iyer S.S., Sadler J.J., Colegio O.R., Tephly L.A., Carter A.B., Rothman P.B., Flavell R.A., Sutterwala F.S. (2008). The Nalp3 inflammasome is essential for the development of silicosis. Proc. Natl. Acad. Sci. USA.

[33] Matsuda T., Yabushita H., Kanaly R.A., Shibutani S., Yokoyama A. (2006). Increased DNA damage in ALDH2-deficient alcoholics. Chem. Res. Toxicol..

[34] Roberts D.W., Churchwell M.I., Beland F.A., Fang J.L., Doerge D.R. (2001). Quantitative analysis of etheno-2'-deoxycytidine DNA adducts using on-line immunoaffinity chromatography coupled with LC/ES-MS/MS detection. Anal. Chem..

[35] Raboisson P, Baurand A, Cazenave J.P., Gachet C., Retat M., Spiess B., Bourguignon J.J. (2002). Novel antagonist sactingat the P2Y(1) purinergicreceptor: Synthesis and conformation alanalysis using potentiometric and nuclear magnetic resonance titration techniques. J. Med. Chem..

[36] Taghizadeh K., McFaline J.L., Pang B., Sullivan M., Dong M., Plummer E., Dedon P.C. (2008). Quantification of DNA damage products resulting from deamination, oxidation and reaction with products of lipid peroxidation by liquid chromatography isotope dilution tandem mass spectrometry. Nat. Protoc..

[37] Kasai H., Nishimura S. (1984). Hydroxylation of deoxyguanosine at the C-8 position by ascorbic acid and other reducing agents. Nucleic Acids Res..

[38] Delaney J.C., Essigmann J.M. (2008). Biological properties of single chemical-DNA adducts: A twenty year perspective. Chem. Res. Toxicol..

[39] Cadet J., Loft S., Olinski R., Evans M.D., Bialkowski K., Richard Wagner J., Dedon P.C., Møller P., Greenberg M.M., Cooke M.S. (2012). Biologically relevant oxidants and terminology, classification and nomenclature of oxidatively generated damage to nucleobases and 2-deoxyribose in nucleic acids. Free Radic. Res..

[40] Berquist B.R., Wilson D.M. (2012). Pathways for repairing and tolerating the spectrum of oxidative DNA lesions. Cancer Lett..

[41] 41Niles J.C., Wishnok J.S., Tannenbaum S.R. (2006). Peroxynitrite-induced oxidation and nitration products of guanine and 8-oxoguanine: Structures and mechanisms of product formation. Nitric Oxide.

[42] Kamiya H. (2003). Mutagenic potentials of damaged nucleic acids produced by reactive oxygen/nitrogen species: Approaches using synthetic oligonucleotides and nucleotides: Survey and summary. Nucleic Acids Res..

[43] Box H.C., Budzinski E.E., Dawidzik J.B., Wallace J.C., Iijima H. (1998). Tandem lesions and other products in X-irradiated DNA oligomers. Radiat. Res..

[44] Crean C., Uvaydov Y., Geacintov N.E., Shafirovich V. (2008). Oxidation of single-stranded oligonucleotides by carbonate radical anions: Generating intrastrand cross-links between guanine and thymine bases separated by cytosines. Nucleic Acids Res..

[45] Hong H., Cao H., Wang Y., Wang Y. (2006). Identification and quantification of a guanine-thymine intrastrand cross-link lesion induced by Cu(II)/H_2_O_2_/ascorbate. Chem. Res. Toxicol..

[46] Nair J., Godschalk R.W., Nair U., Owen R.W., Hull W.E., Bartsch H. (2012). Identification of 3,N(4)-etheno-5-methyl-2'-deoxycytidine in human DNA: A new modified nucleoside which may perturb genome methylation. Chem. Res. Toxicol..

[47] Knutson C.G., Rubinson E.H., Akingbade D., Anderson C.S., Stec D.F., Petrova K.V., Kozekov I.D., Guengerich F.P., Rizzo C.J., Marnett L.J. (2009). Oxidation and glycolytic cleavage of etheno and propano DNA base adducts. Biochemistry.

[48] Otteneder M.B., Knutson C.G., Daniels J.S., Hashim M., Crews B.C., Remmel R.P., Wang H., Rizzo C., Marnett L.J. (2006). *In vivo* oxidative metabolism of a major peroxidation-derived DNA adduct, M1dG. Proc. Natl. Acad. Sci. USA.

[49] Wang H., Marnett L.J., Harris T.M., Rizzo C.J. (2004). A novel synthesis of malondialdehyde adducts of deoxyguanosine, deoxyadenosine, and deoxycytidine. Chem. Res. Toxicol..

[50] Minko I.G., Kozekov I.D., Harris T.M., Rizzo C.J., Lloyd R.S., Stone M.P. (2009). Chemistry and biology of DNA containing 1,N(2)-deoxyguanosine adducts of the alpha,beta-unsaturated aldehydes acrolein, crotonaldehyde, and 4-hydroxynonenal. Chem. Res. Toxicol..

[51] Kawai Y., Furuhata A., Toyokuni S., Aratani Y., Uchida K. (2003). Formation of acrolein-derived 2'-deoxyadenosine adduct in an iron-induced carcinogenesis model. J. Biol. Chem..

[52] Uchida K., Kanematsu M., Sakai K., Matsuda T., Hattori N., Mizuno Y., Suzuki D., Miyata T., Noguchi N., Niki E. (1998). Protein-bound acrolein: Potential markers for oxidative stress. Proc. Natl. Acad. Sci. USA.

[53] Liu X., Lao Y., Yang I.Y., Hecht S.S., Moriya M. (2006). Replication-coupled repair of crotonaldehyde/acetaldehyde-induced guanine-guanine interstrand cross-links and their mutagenicity. Biochemistry.

[54] Ishino K., Shibata T., Ishii T., Liu Y.T., Toyokuni S., Zhu X., Sayre L.M., Uchida K. (2008). Protein N-acylation: H_2_O_2_-mediated covalent modification of protein by lipid peroxidation-derived saturated aldehydes. Chem. Res. Toxicol..

[55] Eder E., Hoffman C. (1993). Identification and characterization of deoxyguanosine adducts of mutagenic beta-alkyl-substituted acrolein congeners. Chem. Res. Toxicol..

[56] Nair U., Bartsch H., Nair J. (2007). Lipid peroxidation-induced DNA damage in cancer-prone inflammatory diseases: A review of published adduct types and levels in humans. Free Radic. Biol. Med..

[57] Blair I.A. (2008). DNA adducts with lipid peroxidation products. J. Biol. Chem..

[58] Kasai H., Kawai K. (2008). 4-oxo-2-hexenal, a mutagen formed by omega-3 fat peroxidation: Occurrence, detection and adduct formation. Mutat. Res..

[59] Salomon R.G., Hong L., Hollyfield J.G. (2011). Discovery of carboxyethylpyrroles (CEPs): Critical insights into AMD, autism, cancer, and wound healing from basic research on the chemistry of oxidized phospholipids. Chem. Res. Toxicol..

[60] Zhong W., Hee S.Q. (2004). Quantitation of normal and formaldehyde-modified deoxynucleosides by high-performance liquid chromatography/UV detection. Biomed. Chromatogr..

[61] Wang M., Cheng G., Balbo S., Carmella S.G., Villalta P.W., Hecht S.S. (2009). Clear differences in levels of a formaldehyde-DNA adduct in leukocytes of smokers and nonsmokers. Cancer Res..

[62] Matsuda T., Matsumoto A., Uchida M., Kanaly R.A., Misaki K., Shibutani S., Kawamoto T., Kitagawa K., Nakayama K.I., Tomokuni K. (2007). Increased formation of hepatic *N*^2^-ethylidene-2'-deoxyguanosine DNA adducts in *aldehyde dehydrogenase 2*-knockout mice treated with ethanol. Carcinogenesis.

[63] Olsen R., Molander P., Øvrebø S., Ellingsen D.G., Thorud S., Thomassen Y., Lundanes E., Greibrokk T., Backman J., Sjöholm R. (2005). Reaction of glyoxal with 2'-deoxyguanosine, 2'-deoxyadenosine, 2'-deoxycytidine, cytidine, thymidine, and calf thymus DNA: Identification of DNA adducts. Chem. Res. Toxicol..

[64] Frischmann M., Bidmon C., Angerer J., Pischetsrieder M. (2005). Identification of DNA adducts of methylglyoxal. Chem. Res. Toxicol..

[65] Masuda M., Suzuki T., Friesen M.D., Ravanat J.L., Cadet J., Pignatelli B., Nishino H., Ohshima H. (2001). Chlorination of guanosine and other nucleosides by hypochlorous acid and myeloperoxidase of activated human neutrophils. Catalysis by nicotine and trimethylamine. J. Biol. Chem..

[66] Asahi T., Kondo H., Masuda M., Nishino H., Aratani Y., Naito Y., Yoshikawa T., Hisaka S., Kato Y., Osawa T. (2010). Chemical and immunochemical detection of 8-halogenated deoxyguanosines at early stage inflammation. J. Biol. Chem..

[67] Byun J., Henderson J.P., Heinecke J.W. (2003). Identification and quantification of mutagenic halogenated cytosines by gas chromatography, fast atom bombardment, and electrospray ionization tandem mass spectrometry. Anal. Biochem..

